# Overexpression of the poplar *NF-YB7* transcription factor confers drought tolerance and improves water-use efficiency in *Arabidopsis*

**DOI:** 10.1093/jxb/ert262

**Published:** 2013-09-04

**Authors:** Xiao Han, Sha Tang, Yi An, Dong-Chao Zheng, Xin-Li Xia, Wei-Lun Yin

**Affiliations:** Nation Engineering Laboratory for Tree Breeding, College of Biological Sciences and Biotechnology, Beijing Forestry University, No. 35 Tsinghua East Road, Beijing, PR China

**Keywords:** *Arabidopsis*, drought tolerance, *NF-YB*, poplar, transcription factor, water-use efficiency.

## Abstract

Water deficit is a serious environmental factor limiting the growth and productivity of plants worldwide. Improvement of drought tolerance and efficient water use are significant strategies to overcome this dilemma. In this study, a drought-responsive transcription factor, NUCLEAR FACTOR Y subunit B 7 (*PdNF-YB7*), induced by osmotic stress (PEG6000) and abscisic acid, was isolated from fast-growing poplar clone NE-19 [*Populus nigra* × (*Populus deltoides* × *Populus nigra*)]. Ectopic overexpression of *PdNF-YB7* (*oxPdB7*) in *Arabidopsis* enhanced drought tolerance and whole-plant and instantaneous leaf water-use efficiency (WUE, the ratio of biomass produced to water consumed). Overexpressing lines had an increase in germination rate and root length and decrease in water loss and displayed higher photosynthetic rate, instantaneous leaf WUE, and leaf water potential to exhibit enhanced drought tolerance under water scarcity. Additionally, overexpression of *PdNF-YB7* in *Arabidopsis* improved whole-plant WUE by increasing carbon assimilation and reducing transpiration with water abundance. These drought-tolerant, higher WUE transgenic *Arabidopsis* had earlier seedling establishment and higher biomass than controls under normal and drought conditions. In contrast, *Arabidopsis* mutant *nf-yb3* was more sensitive to drought stress with lower WUE. However, complementation analysis indicated that complementary lines (*nf-yb3/PdB7*) had almost the same drought response and WUE as wild-type Col-0. Taken together, these results suggest that *PdNF-YB7* positively confers drought tolerance and improves WUE in *Arabidopsis*; thus it could potentially be used in breeding drought-tolerant plants with increased production even under water deficiency.

## Introduction

Poplar is an important tree species of great economic and ecological importance worldwide. It is also one of the fastest growing trees and its high productivity requires a high consumption of water (Ridge *et al.*, 1986; Tschaplinski and Blake, 1989; Zsuffa *et al.*, 1996; Bradshaw *et al.*, 2000; Monclus *et al.*, 2005). Environmental abiotic stress, such as drought, salinity, and cold, could have harmful effects on the development and growth of poplars (Tschaplinski *et al.*, 1994; Renaut *et al.*, 2005; Escalante-Pérez *et al.*, 2009). North China is a mostly dry or semi-dry area. Water resources are key factors in plant yields (Sun *et al.*, 2006). Water-use efficiency (WUE), measured as the biomass produced per unit transpiration, describes the relationship between water use and plant productivity. The basic physiological definition of leaf WUE is equal to the ratio of photosynthesis to transpiration, also referred to as transpiration efficiency (Karaba *et al.*, 2007). High WUE could increase the total production of plants under variable soil water content in the soil as an adaptation to water scarcity.

Water-deficit-inducible genes can be classified into two major groups: the first group encodes function proteins, such as aquaporins, LEA proteins, and chaperones; the second group includes genes encoding regulatory proteins, such as transcription factors, protein kinases, hormones, and other signal molecules (Shinozaki *et al.*, 2003; Valliyodan and Nguyen, 2006). Transcription factors play critical roles in controlling intrinsic developmental processes and responses to external stimuli by influencing the expression of downstream targets and have been confirmed to improve drought resistance in transgenic plants (Shinozaki *et al.*, 2003). The transcription factors involved in drought-responsive pathways are distributed mainly in the *AP2/ERF*, *bZIP*, *NAC*, *MYB*, *C2H2* zinc finger, and *WRKY* families (Abe *et al.*, 1997; Kizis *et al.*, 2001; Sakamoto *et al.*, 2004; Lu *et al.*, 2007; Xiang *et al.*, 2008; Wang *et al.*, 2009). *AtMYB61*, a member of the R2R3-MYB family of transcription factors in *Arabidopsis thaliana*, closes stomata to limit water loss while directing the establishment of water-conducting xylem vessels with larger vessel diameter and root system proliferation to better seek, acquire, and transport water (Romano *et al.*, 2012). Overexpression of *TaWRKY2* and *TaWRKY19* wheat *WRKY* transcription factors could regulate drought stress tolerance in *Arabidopsis* by activating downstream target genes related to drought resistance, such as *DREB*, *RD29*, and *COR6.6* (Niu *et al.*, 2012). In addition, another transcription factor family, *GTL1*, could control stomatal density by transrepression of *SDD1* and negatively regulate WUE and drought tolerance in *Arabidopsis* (Yoo *et al.*, 2010).

Recently, the NUCLEAR FACTOR Y family (NF-Y) binding specifically to the CCAAT box, was identified in drought-responsive pathways and also named for the CCAAT-box binding factor (CBF) (Nelson *et al.*, 2007; Stephenson *et al.*, 2007; Li *et al.*, 2008; Thirumurugan *et al.*, 2008). NF-Y is a ubiquitous nuclear transcription factor that consists of three subunits: NF-YA (CBF-B, HAP2); NF-YB (CBF-A, HAP3); and NF-YC (CBF-C, HAP5). In yeast, the subunits are called HEME ACTIVATED PROTEIN (HAP) 2, 3, and 5, respectively, and are encoded by single genes (Mantovani, 1999). The CCAAT box is a *cis*-acting element widely found in the eukaryotic promoter region, which is bound specifically with proteins encoded by the *NF-Y* family and regulates a series of related gene expressions (Testa *et al.*, 2005). Recent reports elucidate some functions of the *NF-Y* family. Several *NF-YB* and *NF-YC* members in *Arabidopsis*, tobacco, and wheat play roles in light regulation and flowering time (Kumimoto *et al.*, 2008, 2010; Stephenson *et al.*, 2010, 2011; Hackenberg *et al.*, 2012). *AtNF-YB6* (*L1L*) and *AtNF-YB9* (*LEC1*) are involved in embryo development in seeds (Kwong *et al.*, 2003; Yamamoto *et al.*, 2009). In recent years, *NF-Y* family members in plants have been found to function in drought stress. Li *et al.* (2008) found that the plants overexpressing *AtNF-YA5* display reduced stomatal aperture and leaf water loss and significantly promote drought resistance and *AtNF-YA5* is regulated transcriptionally by abscisic acid (ABA) and posttranscriptionally by *miR169*. The overexpression of *AtNF-YB1* confers improved performance in *Arabidopsis* with higher water potential and photosynthesis rates under drought treatments. Transgenic maize plants with *ZmNF-YB2*, the homologue of *AtNF-YB1*, show drought tolerance based on increased chlorophyll content, stomatal conductance, and photosynthesis rates and decreased leaf temperature under drought conditions and exhibit a grain yield advantage (Nelson *et al.*, 2007). However, little has been reported about the relationship between *NF-Y* transcription factors and WUE.

This study identified a poplar drought-responsive *NF-Y* family member from the fast-growing black cottonwood with high WUE, which conferred drought tolerance and improved plant WUE under water deficit.

## Materials and methods

### Plant materials and growth conditions

The poplar genotype NE-19 [*Populus nigra* × (*Populus deltoides* × *Populus nigra*)] was used in this study. NE-19 cuttings with 15-cm-long stems were planted in April 2010, in the nursery of Beijing Forestry University, Beijing, China (40° 0′ 7.05′′ N 116° 15′ 1.60′′ E) for further gene analysis.

*Arabidopsis* Col-0 was selected as the wild-type control. *Arabidopsis* mutant *nf-yb3* (stock name SALK_074951) was ordered from the *Arabidopsis* Biological Resource Center and the homozygous mutant for T-DNA insertion within *AtNF-YB3* (AT4G14540) was verified by PCR. *Arabidopsis* seeds were sterilized by a 60-s 70% ethanol treatment followed by 1% NaClO within 10min and four washes in distilled water. Seeds were sown on half-strength Murashige and Skoog (MS) plates with 3% sucrose and 0.6% agar and stratified for 2 d at 4 °C before being transferred to the culture room at 22 °C under a 16/8 light/dark cycle. After germination, 10-d-old *Arabidopsis* seedlings were transplanted and grown at a density of four plants per 7×7 × 6.5cm pot containing a mixture of soil and vermiculite (2:1) at 22 °C under a 16/8 light/dark cycle (150 μmol m^−2^ s^−1^ and 70% relative humidity.

### Poplar gene cloning, transformation, and expression analysis

Total RNA was extracted from the leaves of poplar NE-19 seedlings using the CTAB reagent method described by Chang *et al.* (1993). First-strand cDNA synthesis was performed using M-MLV Reverse Transcriptase and an oligo (dT) primer (Promega, Madison, WI, USA) according to the manufacturer’s instructions (Xing *et al.*, 2011). The *PdNF-YB7* cDNA sequence was amplified by PCR using the primers PdNFYB7f and PdNFYB7r (Supplementary Table S1, available at *JXB* online).

To obtain *35S:PdNF-YB7* and *nf-yb3/PdB7* transgenic plants, the *PdNF-YB7* cDNA was cloned into the pCAMBIA-1304 binary vector under the control of the cauliflower mosaic virus (CaMV) 35S promoter and transformed into *Arabidopsis* Col-0 and mutant lines respectively by the floral dip method (Bechtold *et al.*, 2003) using *Agrobacterium tumefaciens* GV3101. The transgenic lines were identified using half-strength MS plates containing 100mg l^−1^ hygromycin.

For promoter expression analysis, the *PdNF-YB7*pro:GUS construct, including a 2.3-kb fragment upstream from the initiation codon extracted from poplar NE-19 genomic DNA, was cloned into the pBI121 vector and transformed into *Arabidopsis* Col-0.

For subcellular localization of *PdNF-YB7* in plant cells, GFP fusion proteins were observed using a confocal laser scanning microscope (DMI6000 CS; Leica, Wetzlar, Germany).

To analyse the expression levels of related genes, total RNA was extracted from transgenic, wild type, mutant, and complementation plants by the CTAB method. Real-time PCR analysis was performed using primers PdB7 and PdActin (Supplementary Table S1). Quantitative real-time PCR (qPCR) analysis followed the procedure described by Chen *et al.* (2009). SYBR Green was used to monitor the kinetics of PCR product formation in qPCR. The 18S rRNA transcript, as an internal control, was used to quantify the relative expression levels of genes in samples. The primer sequences are shown in Supplementary Table S2.

### Histochemical staining analysis

To test the induction of GUS expression by osmotic stress, 10-d-old *Arabidopsis* seedlings were transferred from half-strength MS plates to half-strength MS liquid medium containing 25mM PEG6000 or 200mM mannitol for osmotic treatment. The controls were treated with half-strength MS liquid medium. GUS staining was performed by incubating the plants in GUS solution containing 100mM Na_2_HPO_4_ buffer, 1mM K_3_(Fe[CN]_6_), 1mM K_2_(Fe[CN]_6_), 10mM EDTA, 1% (v/v) Triton X-100 and 0.5mg ml^–1^ 5-bromo-4-chloro-3-indolyl-β-d-glucuronic acid overnight at 37 °C in the dark, followed by clearing with 75% ethanol for another hour.

### Physiological experiments

Three independent batches of seeds were used to confirm the germination rate. Twenty seeds for a line in one batch were used for germination comparison between *oxPdB7s*, Col-0, *nf-yb3* and *nf-yb3/PdB7* plants. Based on the diversity of germination time, the seeds would be separately sown on the plates to unify germination time. The *Arabidopsis* lines after germination were grown vertically for 8 d and the primary root length was measured. Rosette leaves were removed from 18-d-old seedlings grown in the soil and the leaf area was computed using Photoshop (Adobe Systems, San Jose, CA, USA). After transferring the plants to soil, plant height was measured every 3 d during the bolting period to calculate the average stem elongation rate.

### Analysis of WUE

Instantaneous leaf WUE was defined as the ratio of the rate of CO_2_ assimilation (photosynthetic rate)/transpiration rate (Wong *et al.*, 1978), which were measured using the Li-6400 Portable Photosynthesis System (Li-Cor, Lincoln, NE, USA). The young, fully expanded rosette leaves of 3-week-old plants were measured at an ambient CO_2_ concentration of 400 μmol mol^−1^, photosynthetic photon flux density of 600 μmol m^−2^ s^−1^, and a chamber temperature of 22 °C. Whole-plant WUE was measured by gravimetric analysis (Xing *et al.*, 2011). Plants were cultured in pots of known weight filled with soil at saturated field capacity. During the experimental period, the pots were weighed daily (0.1g accuracy) and the difference in weight on subsequent days was corrected by adding water to maintain the saturated field capacity. Additionally, filled pots without plants were used to calculate water loss by evaporation. The water added during the experimental period from 22 to 42 d after germination was summed as the cumulative water transpired (CWT) minus evaporation. Representative plants from *oxPdB7#1*, *#2*, *#3*, Col-0, *nf-yb3*, and *nf-yb3/PdB7* were sampled to measure the initial biomass (total biomass including rosette, inflorescence and root) at 22 d after germination (B22). The seedlings were oven-dried for 16h at 70 °C and weighed. The samples from 42 d after germination (B42) were measured as above. The whole-plant WUE was calculated as (B42 − B22)/(CWT between 22 and 42 d after germination).

### Drought experiments

For stress treatment experiments, *Arabidopsis* seeds were sown on half-strength MS plates with 200mM mannitol and germination rates were recorded. The primary root length was compared on half-strength MS plates with 200mM mannitol where *Arabidopsis* seedlings were transplanted after germination on half-strength MS plates. The seedlings were watered for 15 d after transplanted into the soil, and then water was withheld. The pots were put on absorbent paper for a period of 10 d. After 10 d of water deficit, the pots were rewatered. Plant growth was determined 8 d after rewatering. The soil collected at the three stages (well-watered, drought, rewatered) were oven-dried to a constant weight for 16h at 90 °C and weighed for measurement of soil water content. The photosynthetic rates, instantaneous leaf WUE, and leaf water potential were measured at the three stages. The measurements of photosynthetic rates, instantaneous leaf WUE, and total biomass were described as previously. The leaf water potential was measured *in situ* nondestructively at the leaf surface of *Arabidopsis* seedlings using psychrometers (L-51A; WESCOR, Utah, USA) connected to the PSYPRO Water Potential System (WESCOR).

### Water loss measurements

Rosette leaves of *oxPdB7s*, Col-0, *nf-yb3*, and *nf-yb3/PdB7* plants, which were grown under normal conditions for 25 d after germination, were excised, weighed immediately (leaves weighing approximately 1g were harvested and used immediately for experiments), and incubated on a bench at room temperature and at 70% humidity and 150 μmol m^−2^ s^−1^. Losses in fresh weight were monitored at the times indicated (Ma *et al.*, 2010). Water loss is expressed as the percentage of initial fresh weight.

## Results

### Identification and molecular characterization of differentially expressed genes

According to the microarray profile analysis of *Populus euphratica* response to drought stress (Yan *et al.*, 2012a), several *NF-YB* family genes are induced by drought. Further qPCR analysis validated that *PeNF-YB7* was especially upregulated in the leaves of drought-stressed poplars (Gray *et al.*, 2009; Cao *et al.*, 2011; Yan *et al.*, 2012b). To study the role of the drought-related gene *NF-YB* in *P. nigra* × (*P. deltoides* × *P. nigra*), the current study characterized the poplar NUCLEAR FACTOR Y subunit B 7 (*PdNF-YB7*) (GenBank accession KC460319), the homologue of *P. euphratica PeNF-YB7*, for future research. The *PdNF-YB7* cDNA is 672bp in length and encodes 223 amino acid residues with a predicted molecular mass of 24.644kDa and an isoelectric point of 7.56.

**Fig. 1.**
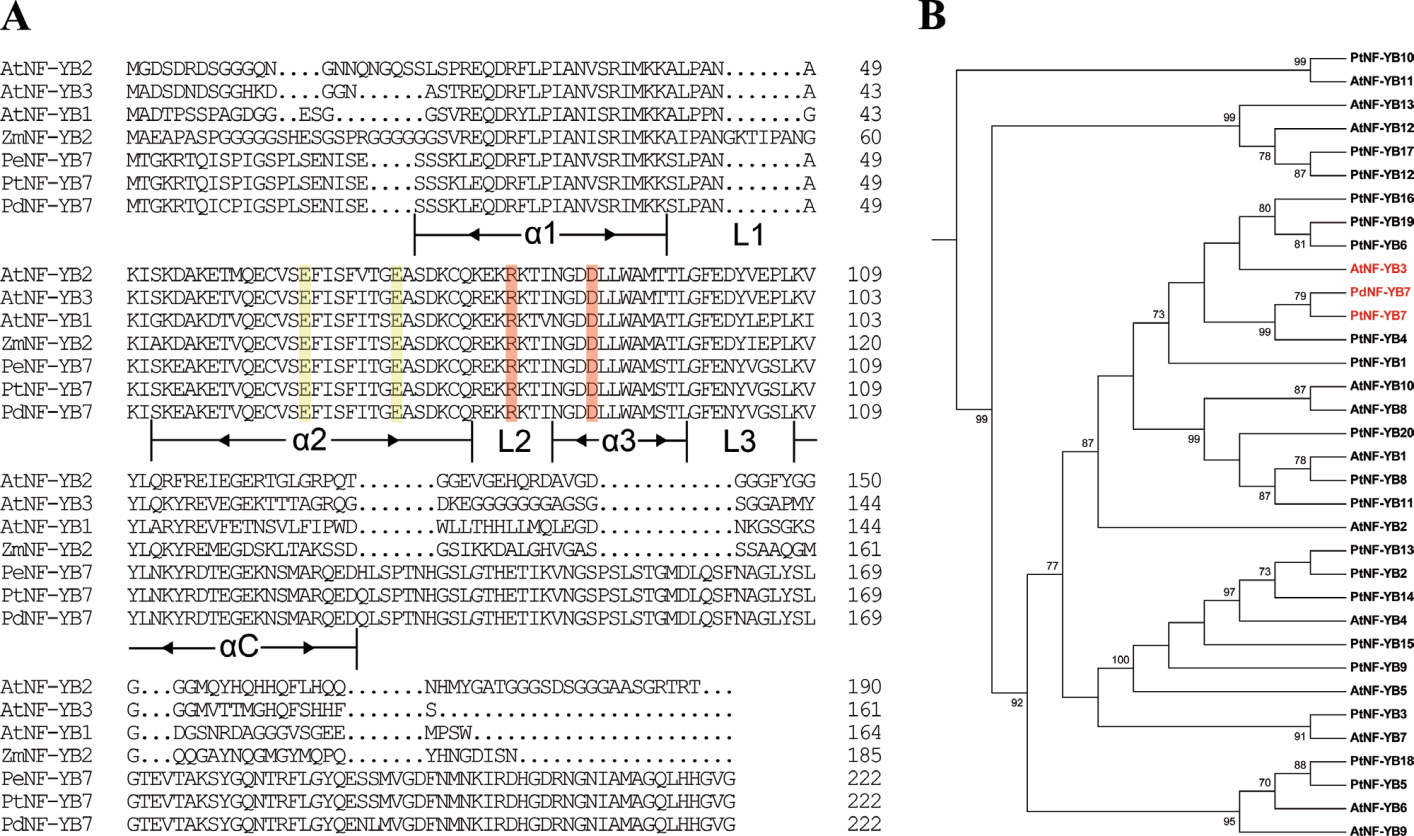
The *NF-YB7* gene of *P. nigra* × (*P. deltoides* × *P. nigra*). (A) Multiple alignment of amino acid sequences of PdNF-YB7 and other plant NF-YBs. AtNF-YB1 (AT2G38880), AtNF-YB2 (AT5G47640), and AtNF-YB3 (AT4G14540) sequences were obtained from the TAIR database and ZmNF-YB2 (DQ333305), PeNF-YB7 (HQ161880), and PtNF-YB7 (POPTR_0007s06480) from NCBI and PopGenIE. The helix motifs are underlined in black (α1, α2, α3, and αC) and the loops are between two helixes (L1, L2, and L3). The Arg and Asp residues, highlighted in red, function in the combination of NF-YB and NF-YC. The two Glu residues, highlighted in yellow, function in the junction with NF-YA. (B) Phylogenetic relationships between poplar and *Arabidopsis* NF-YB family members. The Phylogeny.fr online web service was used for analysis of phylogenetic relationships. The *Arabidopsis* NF-YB family sequences were obtained from the TAIR database and the poplar NF-YB family sequences from the PopGenIE database.

The protein structure alignment using InterPro (http://www.ebi.ac.uk/interpro/) showed that the *PdNF-YB7* sequence domain includes a NF-YB transcription factor conserved site (IPR003956), NF-YB binding site (IPR003957), and NF-YB archaeal histone (IPR003958) (Supplementary Fig. S1). The results indicate that *PdNF-YB7* is a member of the NF-YB transcription factor family. Multiple sequence alignment revealed that the *PdNF-YB7* secondary structure has a basic helix−loop−helix motif composed of four helices and three loops as in NF-YB family conserved domains (Fig. 1A). This highly conserved domain plays a central role in the junction between the NF-Y transcription factor and DNA and interaction between NF-Y and proteins (Mantovani, 1999). Additionally, the Arg in loop 2 and Asp in helix 3, which function in combinations of NF-YB and NF-YC, are highly conserved in the *PdNF-YB7* protein. The two Glu in helix 2 are important for NF-YA binding (Romier *et al.*, 2003). The phylogenetic relationship between the poplar and *Arabidopsis* NF-YB family members was further analysed by amino acid sequence alignment (Dereeper *et al.*, 2008, 2010). As shown in Fig. 1B, PdNF-YB7 did not cluster with known-function NF-YB proteins from *Arabidopsis*, such as drought-resistance protein, AtNF-YB1 (Nelson *et al.*, 2007), and seed development-related proteins AtNF-YB9 and AtNF-YB6 (Kwong *et al.*, 2003; Yamamoto *et al.*, 2009). The results suggest that *PdNF-YB7* differs from known-function *NF-YB* genes in *Arabidopsis* and has a specific function in poplars. The closest homologue to PdNF-YB7 was poplar protein PtNF-YB7 (NF-YB7 for *Populus trichocarpa*), which exhibits 99% identity. Moreover, this study found that, although not entirely orthologous to *PdNF-YB7* (*PtNF-YB6*, *PtNF-YB19*, and *PtNF-YB16* are even closer), *AtNF-YB3* has the closest relationship to *PdNF-YB7* among the *Arabidopsis NF-YB* family members using *PdNF-YB7* as a phylogenetic control. For this reason, *nf-yb3* was chosen for complementation experiments.

### Expression pattern of poplar PdNF-YB7

**Fig. 2.**
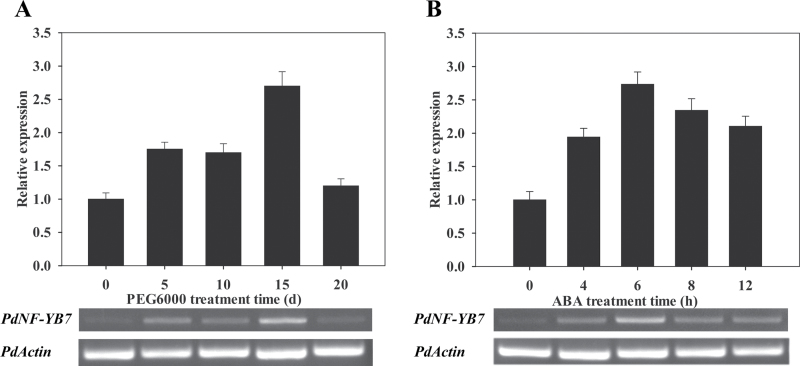
Expression of *PdNF-YB7* in response to osmotic stress and ABA in poplar. qPCR and PCR assay of accumulation of *PdNF-YB7* transcripts in response to 30% PEG6000 (A) and 200 μM ABA (B). The expression levels were normalized to that of *PdActin*, and the level of *PdNF-YB7* transcript in the control was sent at 1.0. Data are mean ± SE (*n* = 3 experiments).

To investigate the involvement of *PdNF-YB7* in poplar responses to osmotic stress, the expression level of *PdNF-YB7* under 30% PEG6000 was tested by PCR (Yan *et al.*, 2012b). The results indicate that the expression level of *PdNF-YB7* rose gradually with increased stress intensity and peaked at 15 d of PEG treatment (Fig. 2A). ABA is an important secondary signalling molecule and its exogenous application can cause similar effects to osmotic stress and mediate some drought-responsive genes (Zhu, 2002). Thus, 200 μM ABA treatment was used to test the response of *PdNF-YB7* in poplar (Chen *et al.*, 2009). The expression of *PdNF-YB7* increased ~2.7-fold by 6h, and then decreased slightly after 6h (Fig. 2B). The results indicate that ABA mainly functions in the early stage of osmotic stress. ABA-responsive stress signalling first modifies the constitutively expressed transcription factors, resulting in the expression of early response genes and then activates downstream stress tolerance effector genes. The early response genes typically encode transcription factors (Zhu, 2002).

**Fig. 3.**
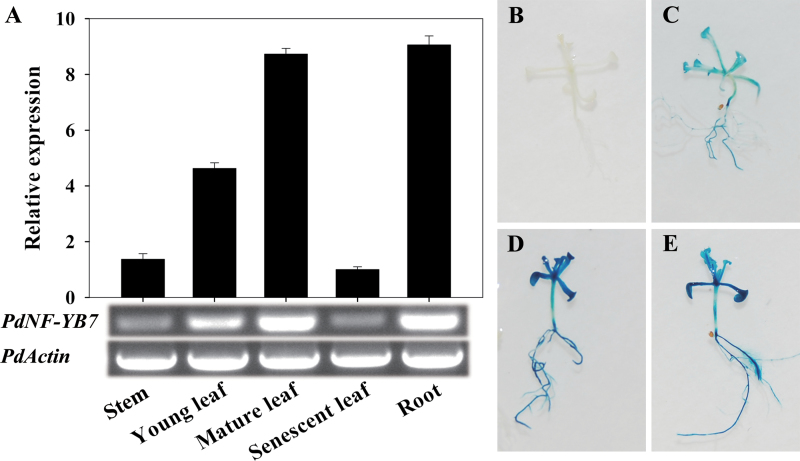
Expression patterns of *PdNF-YB7*. (A) Tissue pattern of *PdNF-YB7* in poplar. Total RNA was isolated from various tissues of seedlings grown in culture. The expression levels were normalized to that of *PdActin*. Data are mean ± SE (*n* = 3 experiments). (B–E) *PdNF-YB7pro*:*GUS* expression patterns in *Arabidopsis*: wild-type control Col-0 (B); control transgenic seedling (C); transgenic seedling treated with 25mM PEG6000 for 3h (D); transgenic seedling treated with 200mM mannitol for 3h (E) (this figure is available in colour at *JXB* online).

To study the tissue-specific presentation of expression of *PdNF-YB7* in poplar, the expression of *PdNF-YB7* was detected in roots, stems, young leaves, mature leaves, and senescent leaves of NE-19 under normal growth conditions. The results showed that *PdNF-YB7* was expressed more highly in mature leaves, young leaves, and roots than in stems and senescent leaves (Fig. 3A).

To assess the expression pattern of *PdNF-YB7*, this study cloned the promoter of *PdNF-YB7* from poplar genomic DNA. A predicted analysis of the *PdNF-YB7* promoter using the PlantCARE database (http://bioinformatics.psb.ugent.be/webtools/plantcare/html/) revealed a series of water-related and abiotic stresses responsive elements, including ABRELATERD1, ACGTATERD1, CBFHV, DPBFCOREDCDC3, MYB, and MYC (Supplementary Table S3). The results suggest that *PdNF-YB7* plays roles in the response to environmental stresses and in plant growth and development.

To analyse the regulatory activity of *PdNF-YB7* during the stress response, this study constructed the *PdNF-YB7pro*:*GUS* expression vector and transformed Col-0 wild-type *Arabidopsis*. Then 25mM PEG6000 and 200mM mannitol were used to determine the osmotic stress response in transgenic plants. Histochemical staining analysis revealed that *GUS* was expressed in transgenic seedlings and that expression was enhanced in response to drought and osmotic treatment and observed throughout the entire plant (Fig. 3B–E).

**Fig. 4.**
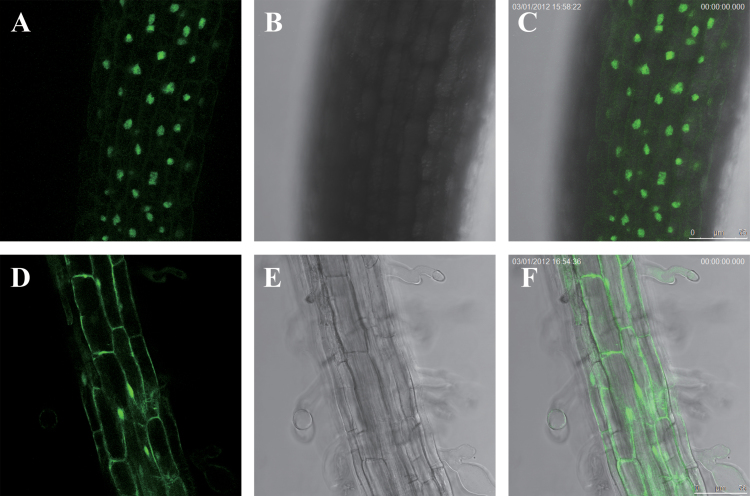
The subcellular localization of *35S:PdNF-YB7-GFP* and control *35S*:*GFP* expression in *Arabidopsis* cells. Cells were detected under a fluorescent or light field by a fluorescence microscope. (A–C) *35S:PdNF-YB7-GFP* seedling in fluorescent light (A), bright light (B), and overlap image of fluorescent and bright light (C). (D–F) Control seedling in fluorescent light (D), bright light (E), and overlap image of fluorescent and bright light (F). Bars, 75 μm (this figure is available in colour at *JXB* online).

To test the subcellular localization of *PdNF-YB7* in plant cells, the *35S:PdNF-YB7-GFP* fusion was constructed and transformed into *Arabidopsis* Col-0. Expression of the *PdNF-YB7*-*GFP* fusion in *Arabidopsis* predominantly accumulated in the nucleus (Fig. 4), consistent with its function as a transcriptional regulator.

### Phenotype of overexpressing lines under well-watered conditions

**Fig. 5.**
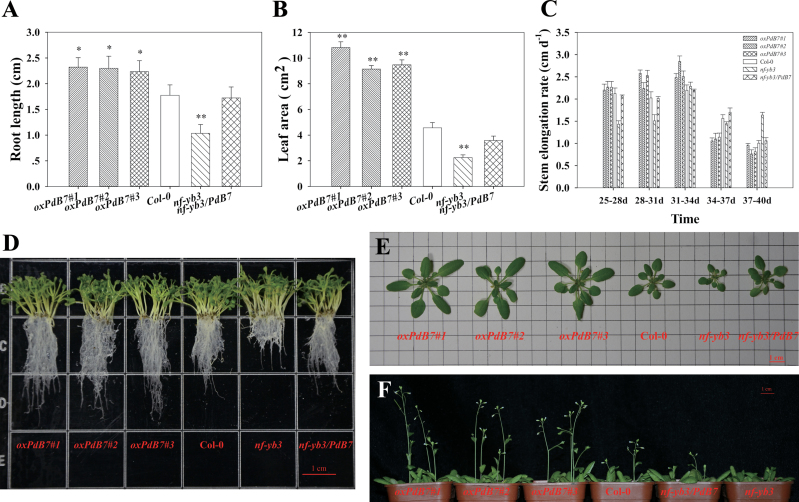
Phenotypes of overexpressing *PdNF-YB7* lines under well-watered conditions. (A) Difference in the primary root length at 8 d after germination. (B) Difference in plant leaf area of 18-d-old seedlings. (C) Plant average stem elongation rate in the time course of growth. (D) Morphological comparisons of the primary root length under half-strength MS for 8 d. (E) Morphology of rosettes of 18-d-old seedlings. (F) Morphology of 23-d-old seedlings grown under well-watered conditions. Data are mean ± SE (*n* = 30). Asterisks denote significant differences: *P ≤ 0.05; **P ≤ 0.01 (this figure is available in colour at *JXB* online).

To evaluate the performance of overexpressing *PdNF-YB7* (*oxPdB7*) lines grown in well-watered conditions, the growth phenotype of overexpressing *Arabidopsis* was observed during different developmental stages. The germination time for *oxPdB7* was 1 or 2 d earlier than the wild-type control Col-0 and mutant *nf-yb3*. Eight days after germination, the primary root length of *oxPdB7* also was longer than Col-0 (1.3-fold) and *nf-yb3* (2.1-fold) (Fig. 5A, D). In 18-d-old seedlings grown in soil, transgenic plants had larger leaves than Col-0 and the mutant (Fig. 5E). The leaf area of *oxPdB7* was about 1.00–1.37-fold larger than Col-0 and 3.07–3.82-fold larger than the mutant (Fig. 5B). The *oxPdB7* lines bolted 22–23 d after germination, whereas the Col-0 and mutant plants bolted at approximately 23–24 d and 24–25 d, respectively. Additionally, the transgenic plants showed higher average stem elongation rate at the earlier shooting stage (25–34 d) compared to Col-0 and mutant (Fig. 5C, F). At 34 d, the inflorescence length of *OxPdB7* varied from 28.2cm to 32.6cm, that of Col-0 varied from 21.3cm to 24.9cm and that of the mutant varied from 15.5cm to 18.3cm. However, at the late stage, Col-0 and *nf-yb3/PdB7* (34–37 d), and *nf-yb3* (37–40 d) displayed higher average elongation rate, respectively (Fig. 5C). The results indicate that the transgenic lines had faster stem elongation at the early shooting stage and earlier seeding establishment.

### Overexpressing PdNF-YB7 improves WUE in Arabidopsis

**Fig. 6.**
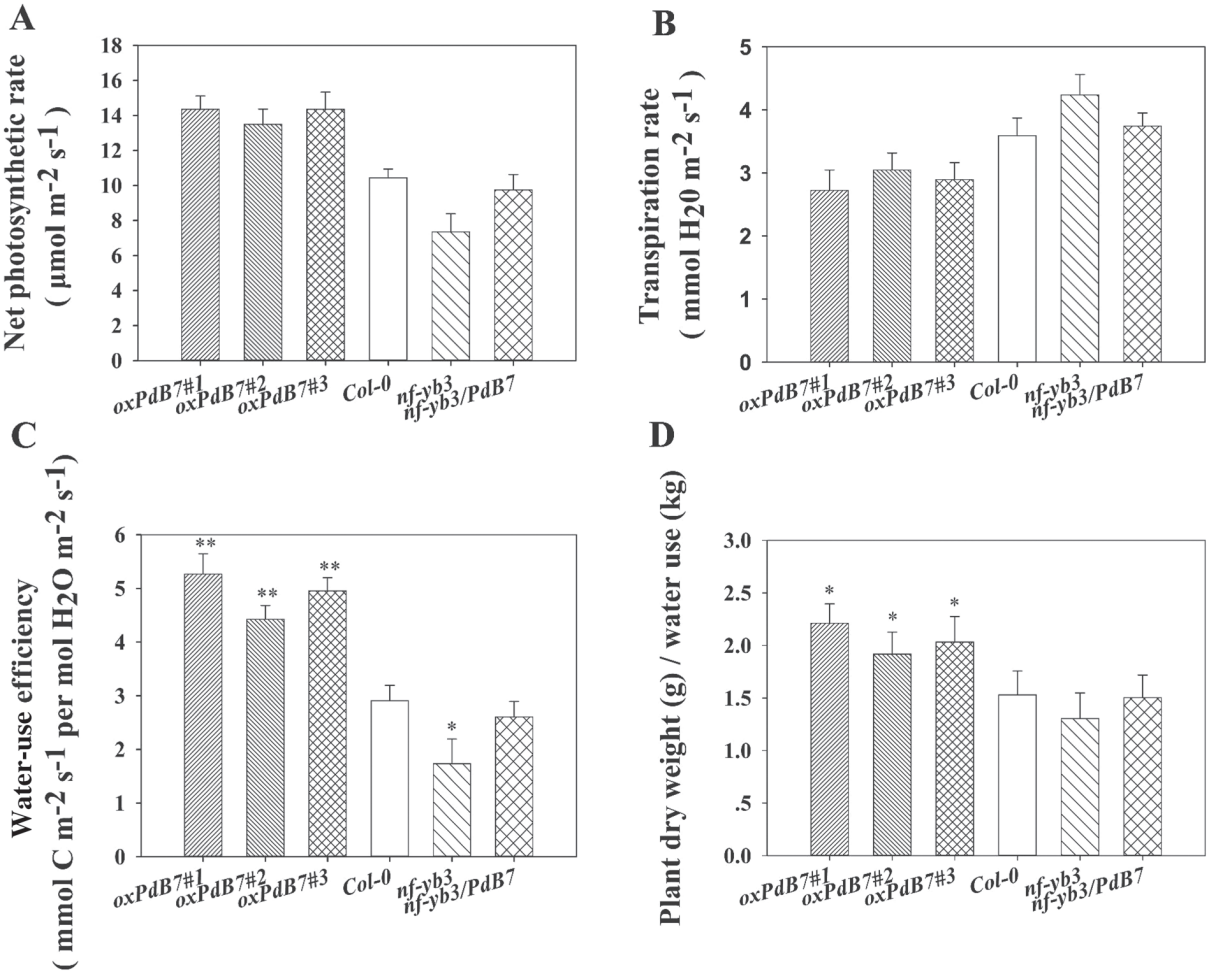
Gas exchange analysis of overexpressing *PdNF-YB7* lines shows that *PdNF-YB7* improved WUE in *Arabidopsis*. (A–C) Net photosynthetic rate (A), transpiration rate (B), and instantaneous leaf WUE (C) of 3-week-old seedlings. (D) Plant dry weight variation under different water consumption (20 d). Data are mean ± SE (*n* = 15). Asterisks denote significant differences: *P ≤ 0.05; **P ≤ 0.01.

The *oxPdB7* lines had higher net photosynthetic rates than Col-0 and the mutant under the same conditions and the mutant, with the slowest net photosynthetic rate, had a rate equivalent to Col-0 after complementation with *PdNF-YB7* (Fig. 6A). For the transpiration rate, the *oxPdB7* lines showed slower rates than Col-0 and the mutant (Fig. 6B). Based on the higher photosynthetic capability and lower transpiration level, the *oxPdB7* lines had higher leaf WUE than Col-0 and the mutant (Fig. 6C). In addition, whole-plant WUE of *oxPdB7* lines was higher than Col-0 and the *nf-yb3* mutant (Fig. 6D).

### Overexpressing PdNF-YB7 increases drought tolerance under water deficit

**Fig. 7.**
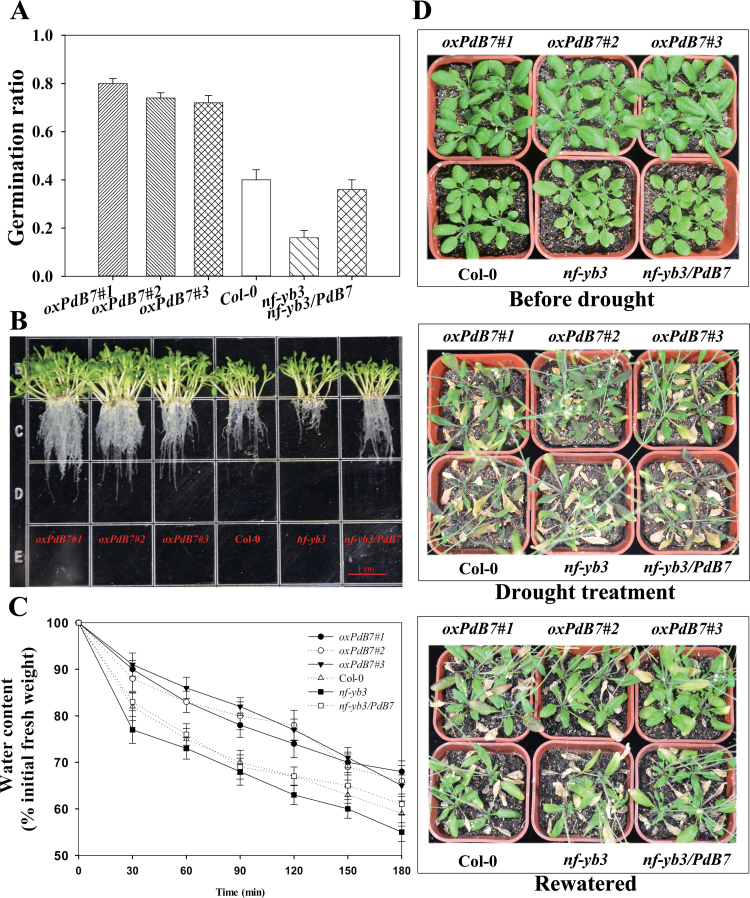
Overexpression of *PdNF-YB7* confers drought tolerance in *Arabidopsis*. (A) Difference in germination ratio among *oxPdB7s*, Col-0, *nf-yb3,* and *nf-yb3/PdB7* plants grown on half-strength MS with 200mM mannitol. (B) Morphological differences in the primary root length of 8-d-old seedlings under 200mM mannitol. (C) Water loss from detached leaves; water loss is expressed as the percentage of initial fresh weight of detached leaves; data are means from five leaves for each of four independent experiments. (D) Morphological differences in drought experiments; the seedlings were grown in soil for 15 d under well-watered conditions; thereafter, water was withheld for 10 d; then plants were rewatered for 8 d. Data are mean ± SE (*n* = 50) (this figure is available in colour at *JXB* online).

To decipher the mechanism by which water deficit affects plant development and growth, various experimental set ups were developed. The seeds of transgenic lines, Col-0, *nf-yb3*, and the complemented line *nf-yb3/PdB7* were sown on half-strength MS culture with 200mM mannitol for osmotic stress. After 4 d, *oxPdB7* lines had more vigorous germination (75.3%) than that of Col-0 (40.4%) and the mutant (16.3%) (Fig. 7A). However, by comparison, all seeds of transgenic lines, Col-0, *nf-yb3*, and *nf-yb3/PdB7* had sprouted after 4 d under normal conditions (data not shown). Additionally, the primary root lengths of 8-d-old seedlings were also different. The *oxPdB7* plants had considerably longer (1.4- and 2.4-fold, respectively) primary roots than that of Col-0 and *nf-yb3* (Fig. 7B). Compared to the primary roots of *Arabidopsis* under well-watered conditions, primary root lengths of *oxPdB7* lines decreased by 21.7% to 27.3% while that of Col-0 decreased by 35.3% and that of *nf-yb3* reduced by 44.8% under drought conditions. Additionally, the detached leaves of transgenic plants lost water more slowly than Col-0 and the mutant (Fig. 7C).

**Fig. 8.**
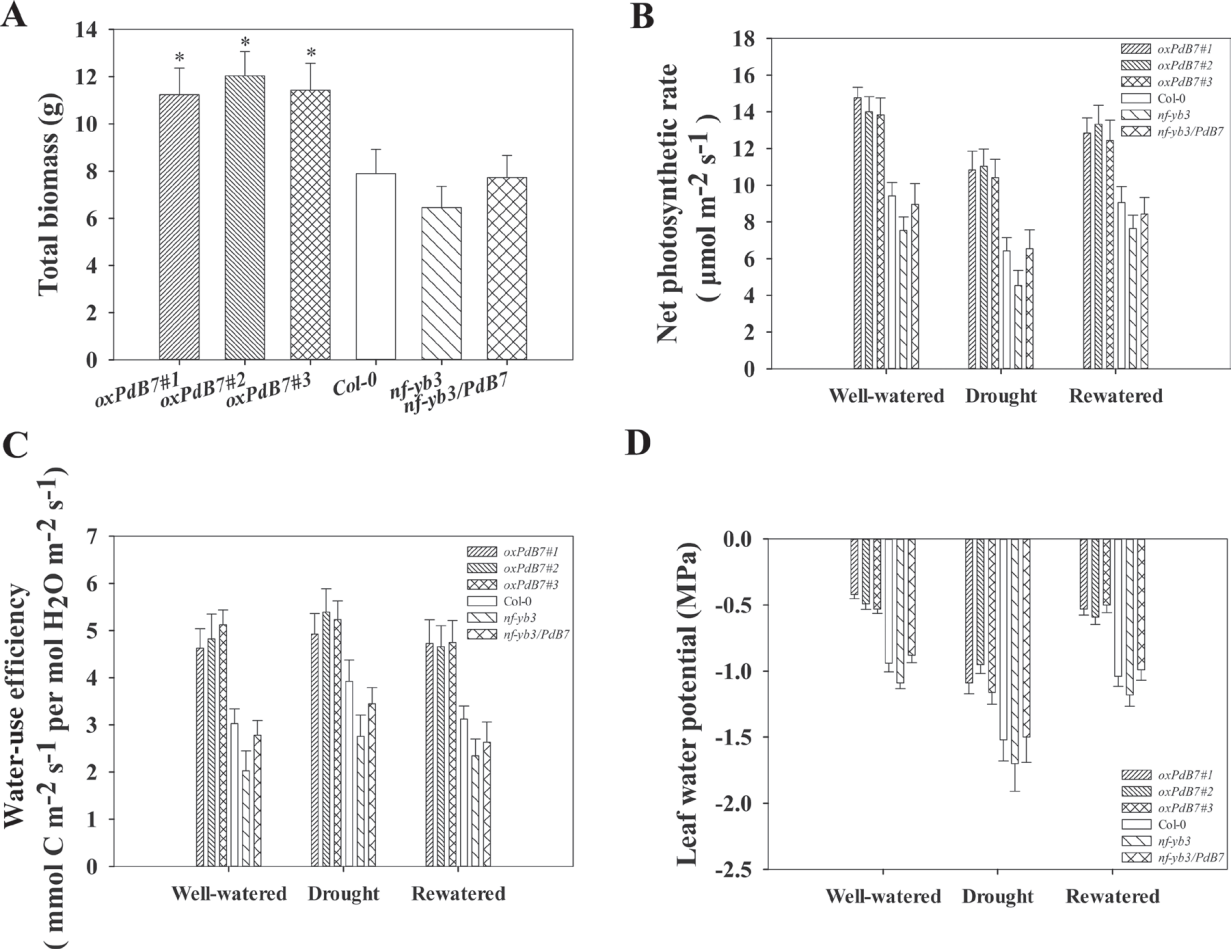
Physiological analysis of overexpressing *PdNF-YB7* lines under drought experiments. (A) Plant total biomass variation for 20 d. (B–D) Net photosynthetic rate (B), instantaneous WUE of leaves under three different stages (C), and leaf water potential (D) under three different stages. Data are mean ± SE (*n* = 15). Asterisks denote significant differences: *P ≤ 0.05.

After the seedlings were transplanted to soil, water deficit was imposed for 10 d, followed by a rewatering period of 8 d (Fig. 7D). Analysis of soil water status for the genotypes explained that soil water contents of three phases in drought experiment were 53.20±1.19% (well-watered), 6.45±1.00% (drought), and 46.01±0.95% (rewatered), respectively. During water deprivation, Col-0 and the *nf-yb3* mutant withered and showed more severe wilting than overexpressing plants, but the *oxPdB7* lines exhibited continued development and growth resulting in more biomass (Fig. 8A). Photosynthesis analysis showed that transgenic lines maintained a significantly higher photosynthetic rate than Col-0 and the mutant under stress treatment (Fig. 8B), resulting in an increase in instantaneous leaf WUE (Fig. 8C). The leaf water potential of *Arabidopsis* seedlings showed significant differences among the transgenic, Col-0, *nf-yb3*, and *nf-yb3/PdB7* seedlings under drought. The transgenic lines had higher leaf water potential compared to Col-0 and the mutant under drought stress (Fig. 8D). Thus, the expression of *PdNF-YB7* was demonstrated to be sufficient to improve tolerance to water scarcity in *Arabidopsis*.

### Expression analysis of stress-responsive genes regulated by the PdNF-YB7 transcription factor

**Fig. 9.**
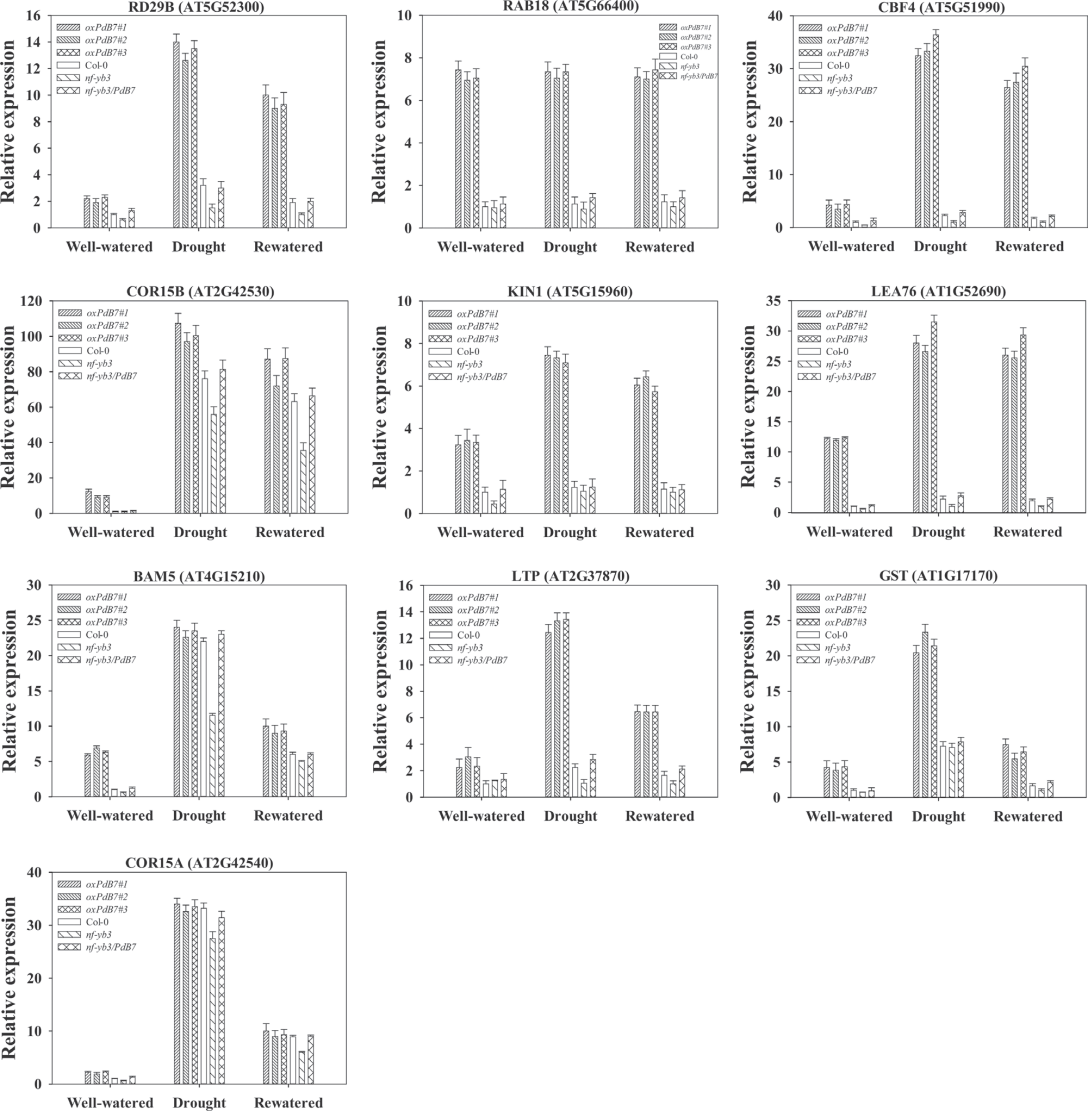
Transcript level analysis of drought-related genes in *oxPdB7s*, Col-0, *nf-yb3*, and *nf-yb3/PdB7* plants. Quantitative real-time PCR was used to analyse expression. The seedlings were sampled on day 14 after being transferred to soil under well-watered conditions on day 6, after drought treatment, and on day 5 after rewatering. *18S rRNA* was used as the internal control. Data are mean ± SE (*n* = 3 experiments).

To determine the improved drought tolerance by altered expression of *PdNF-YB7*, the expression levels of some drought-related genes in the leaves were analysed by qPCR in an independent experiment using the *oxPdB7* lines, Col-0, mutant, and complementation under well-watered, drought, and rewatered conditions. The results showed that ABA pathway markers (*RD29B*, *RAB18*, and *CBF4*), CBF pathway markers (*COR15B*, *KIN1*, and *LEA76*)(Nelson *et al.*, 2007), and several predicted candidate target genes of *AtNF-YA5* (*BAM5*, *LTP*, *GST*, and *COR15A*) (Li *et al.*, 2008) were differentially expressed in the *oxPdB7* lines compared to the *35S*:*NF-YB1* and *35S*:*NF-YA5* lines (Fig. 9). *CBF4*, *COR15B*, *LEA76*, *BAM5*, and *GST* were more highly expressed in the *oxPdB7* lines under well-watered conditions. Under water deficit, these stress-responsive genes were strongly induced in transgenic plants. However, the majority of these genes did not significantly change in *nf-yb3*, suggesting that for many of these genes, *PdNF-YB7* was required for induction by dehydration.

## Discussion

Plants have evolved regulatory mechanisms to adapt to environmental water deficit. Transcription factors regulate expression of the stress-responsive genes by binding specifically to the motif of the promoters to modulate resistance to drought and lower productivity loss (Zhu, 2002; Yamaguchi-Shinozaki and Shinozaki, 2006). NF-Y is an important transcription factor family controlling drought tolerance in plants (Nelson *et al.*, 2007; Stephenson *et al.*, 2007; Li *et al.*, 2008). In a previous study, a drought-responsive *NF-YB* family member *PeNF-YB7* was screened from *P. euphratica* with an Affymetrix Poplar GeneChip microarray and was further identified by qPCR (Yan *et al.*, 2012a). To study drought tolerance of fast-growing poplar, *PdNF-YB7*, the homologue of *PeNF-YB7*, was identified from the high WUE poplar genotype NE-19 [*P. nigra* × (*P. deltoides* × *P. nigra*)] (Yin *et al.*, 2007; Hao *et al.*, 2011). In the current experiments, expression analysis showed that *PdNF-YB7* was differentially expressed and concomitantly induced in response to water deficit in the NE-19 seedlings (Fig. 2A). Overexpression of *PdNF-YB7* in *Arabidopsis* exhibited earlier seedling establishment, longer primary roots, larger leaf areas, and increased photosynthetic rate that conferred drought tolerance and improved WUE in transgenic plants.

Nuclear factor Y is one of the largest transcription factor gene families in plants. A number of NF-Y proteins have been identified as regulators of drought tolerance in different plant species. *AtNF-YB1*, *ZmNF-YB2*, and *TaNF-YB2*, were reported to confer drought resistance in *Arabidopsis*, maize, and wheat, respectively, and to increase crop productivity under drought field tests (Nelson *et al.*, 2007; Stephenson *et al.*, 2007). *PdNF-YB7* was inferred to have the same effect on *Arabidopsis* because of the relatively close evolutionary relationship and genetic distance (Yan *et al.*, 2012b). In addition, another *NF-Y* subunit family member, *AtNF-YA5*, plays a role in drought resistance in *Arabidopsis* (Li *et al.*, 2008). Apart from these, other *NF-YB* family members, such as *AtNF-YB9* (*LEC1*) and *AtNF-YB6* (*L1L*), are essential factors controlling embryonic development and are phylogenetically and functionally distinct from other *NF-YB* family members, such as *AtNF-YB1* and *PtNF-YB7* (Kwong *et al.*, 2003; Yamamoto *et al.*, 2009; Yan *et al.*, 2012b). Most of the above findings were reported for herbaceous plants such as *Arabidopsis* and maize. However, reports of *NF-YB*s in woody plants are still sparse. The *PdNF-YB7* gene studied here is the first reported *NF-YB* gene in fast-growing black poplar.

According to the phylogenetic analysis, *PdNF-YB7* shared high sequence similarity and clustered with a member of the poplar *NF-YB* family genes. The conserved domain analysis of the multiple sequence alignment showed that *PdNF-*YB7, PtNF-YB7, and AtNF-YB3 are very highly conserved. In *Arabidopsis*, *AtNF-YB3* plays an important role in the promotion of flowering specifically under inductive long-day photoperiodic conditions. Consistent with this, the overexpression of *PdNF-YB7* in *Arabidopsis* caused earlier seedling germination time and enhanced the development of both vegetative and reproductive organs (Fig. 5F). Notably, in these experiments, the transcript levels of *PdNF-YB7* were upregulated by drought stress, resembling those of *AtNF-YB1* in *Arabidopsis* (Nelson *et al.*, 2007). Different from *AtNF-YB1*, transcript levels of *PdNF-YB7* were also affected by ABA treatment. Promoter expression analysis of *PdNF-YB7* provided further support for its role in stress tolerance; GUS gene expression was enhanced by drought and was observed throughout the entire plant after stress treatment. Element analysis of the promoter indicated that several ABA-responsive elements were included in *PdNF-YB7* promoter region (Supplementary Table S3). This is the same result of *AtNF-YA5* in *Arabidopsis*; two ABA-responsive element sequences could be found in the promoter region of *AtNF-YA5*, which is proved to be involved in drought resistance (Li *et al.*, 2008). Additionally, increasing evidence is being found for NF-YB/bZIP interactions, and bZIP proteins are well known to be involved in ABA signaling (Liu and Howell, 2010). Interestingly, a tissue-specific expression analysis indicated another difference between *PdNF-YB7* and *AtNF-YB3*. Previous research revealed that *AtNF-YB3* is expressed more highly in flowers and young leaves, but is absent in roots (Siefers *et al.*, 2009), while the current experiments suggested that *PdNF-YB7* is highly expressed in root. This is consistent with *AtNF-YA5*, overexpression of which is proved to increase drought tolerance in *Arabidopsis* (Li *et al.*, 2008).

To better understand the regulatory mechanisms of drought tolerance conferred by overexpressing *PdNF-YB7*, this study confirmed the expression patterns of genes that may potentially be regulated by *NF-Y*s according to previous research on *AtNF-YB1* and *AtNF-YA5*. For the ABA pathway markers (*CBF4*, *RD29B*, and *RAB19*) or CBF pathway markers (*COR15B*, *KIN1*, and *LEA76*), none of them showed differences in expression between *AtNF-YB1* overexpression plants and controls (Nelson *et al.*, 2007). However, in the current study, *RD29B* and *CBF4* showed significant and consistent differences expression in *35S:PdNF-YB7* plants, indicating that the ABA-dependent dehydration response was regulated by *PdNF-YB7*. Drought-inducible genes encoding functional proteins such as *KIN1*, *LEA76*, *LTP*, and *GST* were also highly expressed in transgenic plants, especially in response to water deficit, suggesting that *PdNF-YB7* potentially increased the accumulation of protective proteins under drought conditions.

Transgenic *Arabidopsis* overexpressing poplar *NF-YB7* showed significantly higher biomass under well-watered and drought conditions. Increased photosynthetic leaf area enhanced carbon assimilation, resulting in more biomass accumulation. Several studies have implicated *NF-Y* in controlling photosynthesis by regulating the chloroplast ATP synthase (Kusnetsov *et al.*, 1999) and some nuclear-encoded photosynthesis genes such as *RBCS* and *CAB* (Miyoshi *et al.*, 2003). Transgenic wheat with *TaNF-YB3* has a significant enhancement in leaf chlorophyll content and photosynthesis rate (Stephenson *et al.*, 2011).

Recent studies have shown that root growth is closely connected with drought tolerance (Pennisi, 2008). In the current study, overexpressing *PdNF-YB7* in *Arabidopsis* increased primary root length that led to expansion of the root surface area. Ballif *et al.* (2011) also found that overexpressing *AtNF-YB2* enhanced primary root elongation due to a faster cell division and/or elongation. Additionally, *PdNF-YB7* overexpression transgenic plants also maintained higher leaf water potential than that of wild type under water-deficit conditions. Often during drought conditions, plants avoid low soil water potential by achieving a balance between water absorption and loss, for example, by decreasing the stomatal aperture while maintaining root growth (Verslues *et al.*, 2006). Decrease of stomatal apertures resulted in decreased transpiration rate thus reduced water loss. Meanwhile, increased root length improved absorption of water and mineral solutes. Equality between uptake and loss of water and thereby maintenance of constant leaf water potential is assisted by stomatal changes, which appear to be in response to conditions in the root (Aston and Lawlor, 1979). Altogether, these physiological phenotypes conferred by *PdNF-YB7* support that the gene could be potentially used in breeding drought-tolerant plants and promoting plant production under drought conditions. Further experiments are now needed to characterize the effects of *PdNF-YB7* in poplar to elucidate its regulatory mechanisms in woody plants.

## Supplementary material

Supplementary data are available at *JXB* online.

Supplementary Table S1. Primer sequences used for cloning of *PdNF-YB7* cDNA.

Supplementary Table S2. Primers used for PCR and qPCR.

Supplementary Table S3. Putative elements of the promoter of *PdNF-YB7*.

Supplementary Fig. S1. The structural alignment result of the *PdNF-YB7* protein.

Supplementary Data
