# Supplementary material for: Overexpression of the poplar NF-YB7 transcription factor confers drought tolerance and improves water-use efficiency in Arabidopsis
Source: J Exp Bot. 2013 Sep 4;64(14):4589–601. doi: 10.1093/jxb/ert262 (PMC3808328; doi:10.1093/jxb/ert262)
Supplement: Supplementary Data [file supp_64_14_4589__index.html]

Overexpression of the poplar NF-YB7 transcription factor confers drought tolerance and improves water-use efficiency in Arabidopsis — Overexpression of the poplar NF-YB7 transcription factor confers drought tolerance and improves water-use efficiency in Arabidopsis — Supplementary Data 

# Overexpression of the poplar *NF-YB7* transcription factor confers drought tolerance and improves water-use efficiency in *Arabidopsis*

## Supplementary Data

Data files

**Files in this Data Supplement:**

- Supplementary Data - Supplementary Data
- Supplementary Data - Supplementary Data
