# Supplementary material for: Overexpression of the poplar NF-YB7 transcription factor confers drought tolerance and improves water-use efficiency in Arabidopsis
Source: J Exp Bot. 2013 Sep 4;64(14):4589–601. doi: 10.1093/jxb/ert262 (PMC3808328; doi:10.1093/jxb/ert262)
Supplement: Supplementary Data [file supp_ert262_jexbot094508_file001.pdf]

Journal of Experimental Botany

Overexpression of the poplar *NF-YB7* transcription factor confers drought tolerance and improves water-use efficiency in *Arabidopsis*

Xiao Han[*], Sha Tang[*], Yi An, Dong-Chao Zheng, Xin-Li Xia[†] and Wei-Lun Yin[†]

Table S1. Primer sequences used for cloning of *PdNF-YB7* cDNA, construction of *35S*:*PdNF-YB7*, PCR and qPCR of *PdNF-YB7* in poplar under PEG6000 and ABA treatment. *PdActin* was used as an internal control. Enzyme sites BglII and SpeI were used for construction of the expression vector.

Primer name	Primer sequence
For cloning of *PdNF-YB7* cDNA	
PdNFYB7f	ATGACAGGAAAAAGGACCCAGATC
PdNFYB7r	CCAGCCAACCCCGTGATGAA
For construction of *35S*:*PdNF-YB7*	
rePdNFYB7f	AGATCTGATGACAGGAAAAAGGACCCAGATC
rePdNFYB7r	ACTAGTCCAGCCAACCCCGTGA
For PCR and qPCR	
PdB7f	AAAAGGACCCAGATCTGCCC
PdB7r	TGCATTTGCTGGGAGGGATT
PdActinF	GTCCTCTTCCAGCCATCTC
PdActinR	TTCGGTCAGCAATACCAGG

Table S2. Primers used in qPCR analysis for expression levels of drought-relative genes in *oxPdB7*s, Col-0, *nf-yb3* and *nf-yb3/PdB7* plants.

Gene	Locus ID	Forward Primer	Reverse Primer
RD29B	AT5G52300	ATACCTTCCGACCAGATAGCGG	TACCACCGAGCCAAGAAGTGAC
RAB18	AT5G66400	GCAACTCCACAAGGAAAGTGGT	TGATGACCTGGCAACTTCTCC
CBF4	AT5G51990	AAGCTGCAATGGCGTTTCAG	ACACACCACCATTCTGCTCCTC
COR15B	AT2G42530	TCAGTGGCATGGGTTCTTCTTCCA	GAGGTCATCGAGGATGTTGCCGT
KIN1	AT5G15960	GCAAAACGCGAGCTACCAA	TGTCCAGTCTGTTGCAAGGAGT
LEA76	AT1G52690	AGAACAAGGCACACGATGCAG	AAGTGTGCTTCACCGCATCAG
BAM5	AT4G15210	GGTTGGGTGTTTCCCGGCATCGGA	GGCTTCTCCTAAGATCTGATCTCCA
LTP	AT2G37870	CGTTGAGGCGGCAGGTGAGTGT	TGTAACGTCCACATCGCTTGCCA
GST	AT1G17170	CCTTCCCTCCGATCCTTACAAGA	CAACCCAAGTTTCTTCCTACGTTCG
COR15A	AT2G42540	GTTCTCACTGGTATGGCTTCTT	GTCTTTCGCTTTCTCACCATCTGCT
18S	AT3G41768	CGTCCCTGCCCTTTGTACAC	CGAACACTTCACCGGATCATT

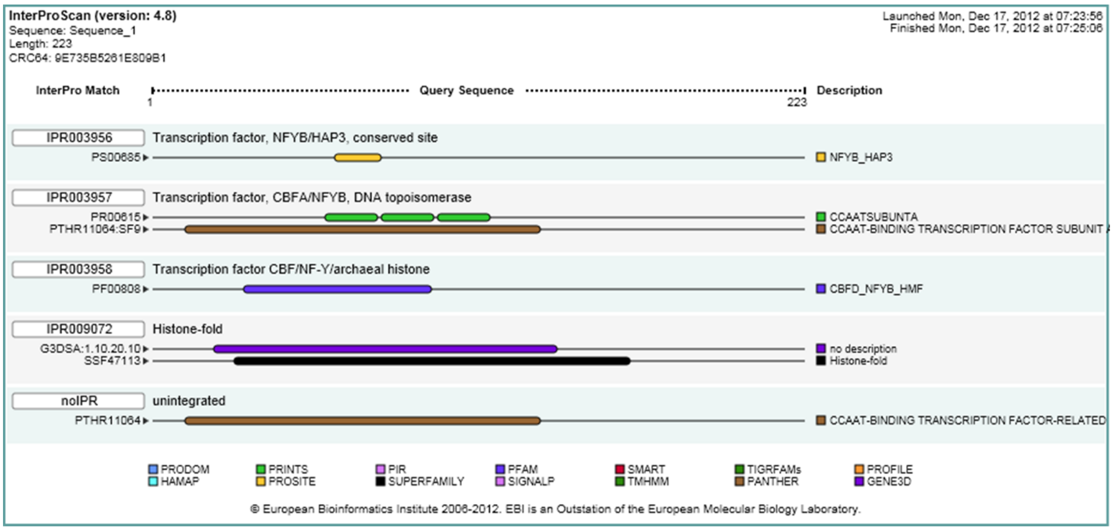

Fig. S1. The structure alignment result of PdNF-YB7 amino acid sequence. The protein structure alignment with InterPro (http://www.ebi.ac.uk/interpro/) showed the PdNF-YB7 sequence domain includes a NF-YB transcription factor conserved site (IPR003956), NF-YB binding site (IPR003957), and NF-YB archaeal histone (IPR003958).

Table S3. Putative elements of the promoter of *PdNF-YB7*. The PlantCARE database (http://bioinformatics.psb.ugent.be/webtools/plantcare/html/) was used for predicted analysis of the *PdNF-YB7* promoter.

Element	Core Sequence	Number	Function
ABRELATERD1	ACGTG	1	Respond to dehydration and dark condition
ACGTATERD1	ACGT	4	Respond to dehydration
ARR1AT	NGATT	32	Respond to CTK
CAATBOX1	CAAT	30	Transcription enhancer
CACTFTPPCA1	YACT	33	Regulation for ppcA1
CATATGGMSAUR	CATATG	2	Respond to auxin
CBFHV	RYCGAC	1	Respond to drought stress
CCAATBOX1	CCAAT	6	Regulation to flowering
CIACADIANLELHC	CAANNNNATC	5	Regulation to circadian rhythm
CPBCSPOR	TATTAG	1	Respond to CTK
CURECORECR	GTAC	10	Respond to copper and oxygen
DPBFCOREDCDC3	ACACNNG	2	Respond to ABA
ERELEE4	AWTTCAAA	1	Respond to ethylene
GAREAT	TAACAAR	1	Respond to GA
GATABOX	GATA	21	Regulation to light
GT1CONSENSUS	GRWAAW	34	Regulation to light
GT1GMSCAM4	GAAAAA	15	Regulation to pathogen and salt
MYB	WAACCA/YAACKG/CNGTTR	10	Regulation to drought and ABA
MYC	CANNTG	20	Regulation to drought, ABA and cold

NTBBF1ARROLB	ACTTTA	2	Respond to auxin
P1BS	GNATATNC	2	Respond to phosphate
POLLEN1LELAT52	AGAAA	23	Regulation to development of pollen
TAAAGSTKST1	TAAAG	9	Regulation to development of stoma
TBOXATGAPB	ACTTTG	4	Regulation to CO_2 fixture
WBOXATNPR1	TTGAC	4	Respond to SA
WBOXHVISO1	TGACT	3	Respond to sugar
