# Supplementary figures and images for: Overexpression of the poplar NF-YB7 transcription factor confers drought tolerance and improves water-use efficiency in Arabidopsis
Source: J Exp Bot. 2013 Sep 4;64(14):4589–601. doi: 10.1093/jxb/ert262 (PMC3808328; doi:10.1093/jxb/ert262)

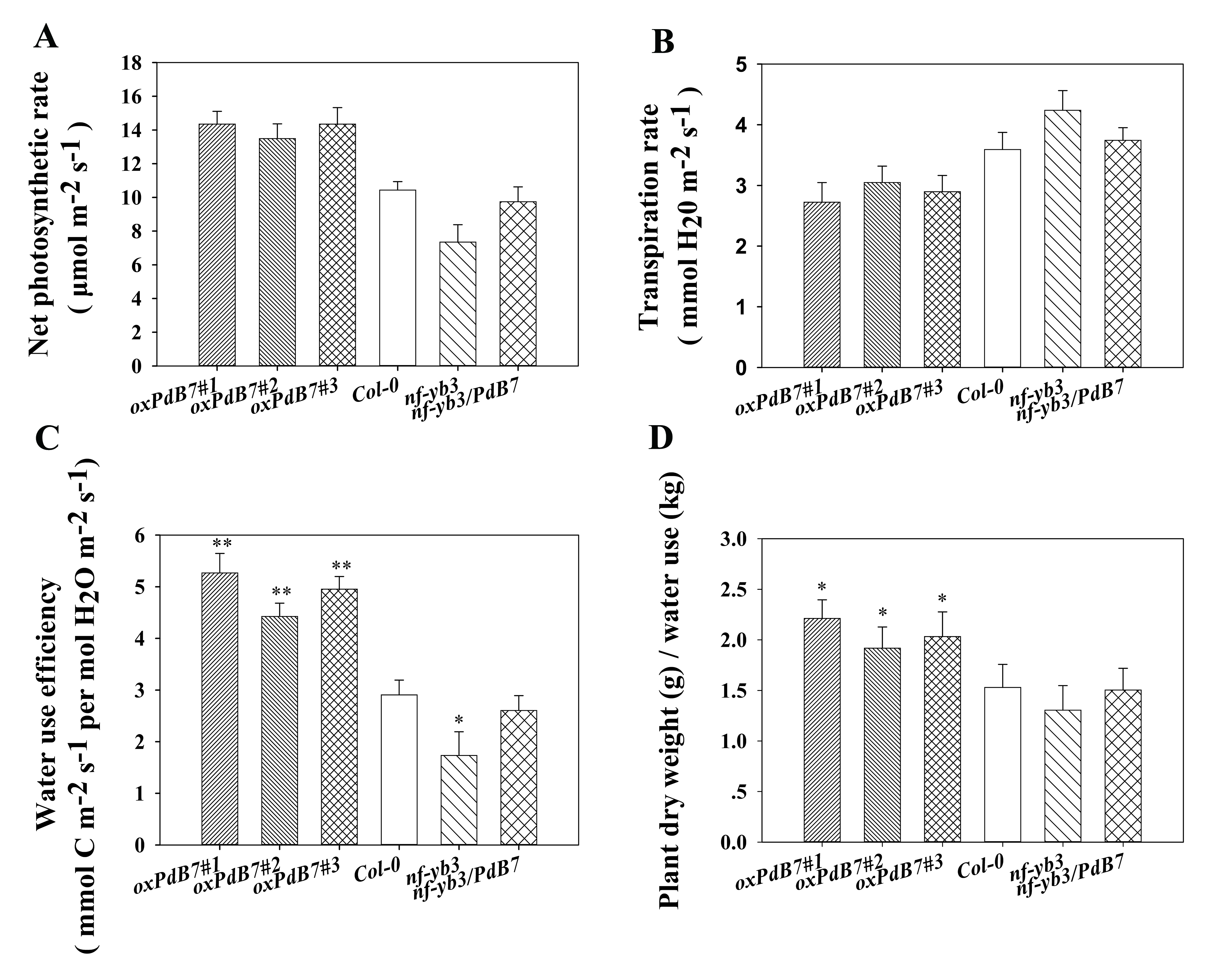

Supplement: Supplementary Data [file supp_ert262_jexbot094508_file002.tif]
